# Time-restricted feeding reduces monocyte production by controlling hematopoietic stem and progenitor cells in the bone marrow during obesity

**DOI:** 10.3389/fimmu.2022.1054875

**Published:** 2022-12-08

**Authors:** Yelim Kim, Youngyoon Lee, Mi Nam Lee, Jiyeon Nah, Narae Yun, Dayong Wu, Munkyong Pae

**Affiliations:** ^1^ Department of Food and Nutrition, Chungbuk National University, Cheongju, Republic of Korea; ^2^ Department of Biological Sciences, Chonnam National University, Gwangju, Republic of Korea; ^3^ Nutritional Immunology Laboratory, Jean Mayer United States Department of Agriculture Human Nutrition Research Center on Aging at Tufts University, Boston, MA, United States

**Keywords:** hematopoietic stem cells, intermittent fasting, myelopoiesis, obesity, time-restricted feeding

## Abstract

Time-restricted feeding (TRF) has emerged as a promising dietary approach in improving metabolic parameters associated with obesity, but its effect on immune cells under obesogenic condition is poorly understood. We conducted this study to determine whether TRF exerts its therapeutic benefit over obesity-induced myeloid cell production by analyzing hematopoietic stem and progenitor cells in bone marrow (BM) and immune cell profile in circulation. Male C57BL/6 mice were fed a low-fat diet (LFD) or high-fat diet (HFD) *ad libitum* for 6 weeks and later a subgroup of HFD mice was switched to a daily 10 h-TRF schedule for another 6 weeks. Mice on HFD *ad libitum* for 12 weeks had prominent monocytosis and neutrophilia, associated with expansion of BM myeloid progenitors, such as multipotent progenitors, pre-granulocyte/macrophage progenitors, and granulocyte/macrophage progenitors. TRF intervention in overweight and obese mice diminished these changes to a level similar to those seen in mice fed LFD. While having no effect on BM progenitor cell proliferation, TRF reduced expression of *Cebpa*, a transcription factor required for myeloid differentiation. These results indicate that TRF intervention may help maintain immune cell homeostasis in BM and circulation during obesity, which may in part contribute to health benefits associated with TRF.

## 1 Introduction

Obesity has become a major health concern over the past decades, a situation often referred to as global pandemic (1, 2). It is increasingly accepted that chronic low-grade inflammation plays an important role in the pathogenesis of obesity and its comorbidities, including type 2 diabetes (3). In this process, macrophages and their circulating precursors, monocytes, have been shown to play critical roles (4, 5). While circulating monocytes (6) and adipose tissue macrophage (ATM) (7, 8) are increased with obesity, the ablation of genes affecting activation (9, 10) or recruitment of myeloid cells (11) could normalize insulin sensitivity in obese insulin resistance animals. It is noteworthy that obesity could promote myelopoiesis in the bone marrow (BM) (12, 13), which in turn potentiate ATM accumulation (12). Thus, normalization of myeloid cell production in BM may be important for uncoupling obesity from metabolic inflammation and the development of insulin resistance.

Time-restricted feeding (TRF) refers to daily limitation in the timing of food intake, usually 10 h or less, followed by a daily fast of at least 14 h (14, 15). Previous studies in rodents and humans reported metabolic benefits of TRF, including reductions in body weight, adiposity, blood pressure, and/or insulin resistance (15–21). We recently demonstrated that TRF intervention reduced numbers of total and proinflammatory ATM, along with an improvement in glucose tolerance under high-fat diet (HFD) feeding (22). Although little is known about the effect of TRF on BM homeostasis under obesogenic conditions, a prior study has shown that lean mice with a fasting exhibit fewer monocytes in circulation by limiting egress from BM (23). Thus, it is worth exploring whether repeated, prolonged fasting on a daily basis by TRF could affect circulating immune cells in overweight and obese mice and regulate obesity-induced myelopoiesis in BM.

Luteolin is a flavonoid widely found in many vegetables and medicinal herbs including broccoli, celery, green peppers, parsley, and perilla leaves and seeds. Luteolin has been reported to have various pharmacological effects, including anti-oxidant (24), anti-inflammatory (25, 26), and antidiabetic activity (27, 28). In addition, luteolin was shown to reduce obesity and obesity-associated inflammation (29–32). Previously, we also demonstrated that luteolin supplementation attenuated ATM inflammation and insulin resistance in postmenopausal obese mice (30). While the beneficial effects of luteolin are recognized, it is not known whether dietary luteolin supplementation in combination of TRF exerts additive effects not only in metabolic parameters but also in BM immune cell composition.

In the current study, we confirmed the therapeutic effects of 10 h-TRF on metabolic markers, including body weight gain and insulin resistance index HOMA-IR in overweight and obese mice. Here, we examined whether TRF initiating after 6 weeks of a HFD *ad libitum* could normalize monocyte populations in mouse blood and BM, along with changes in hematopoietic stem and progenitor pools in the BM. Mechanistically, the BM progenitor cell proliferation and transcription factor expression required for myeloid differentiation were analyzed. In addition, we tested whether luteolin supplementation in combination with TRF could deliver any additional effect on metabolic and immune parameters.

## 2 Materials and methods

### 2.1 Animals

**Figure 1:**
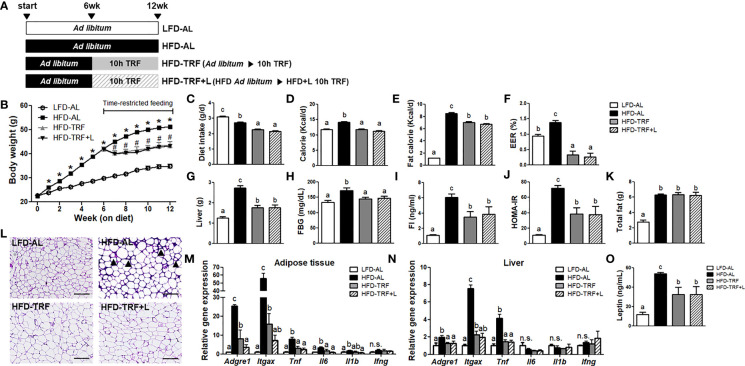
Time-restricted feeding (TRF) reduces body weight gain and insulin resistance index HOMA-IR in obese mice. C57BL/6 male mice (n=14 per group) were given a HFD *ad libitum* starting at 7 weeks of age. After 6 weeks on HFD, the animals were fed for another 6 weeks with HFD *ad libitum* (HFD-AL) or HFD under 10 h time-restricted feeding (HFD-TRF) or HFD plus luteolin under 10 h time-restricted feeding (HFD-TRF+L, luteolin 50 mg/kg diet). The mice fed LFD *ad libitum* (LFD-AL) served as a control. **(A)** Schematic outline of the feeding groups used in this study. **(B)** Average body weight during 12 weeks of experimental period. * *P*<0.05; LFD-AL vs. HFD-AL, ^#^P < 0.05; HFD-AL vs. HFD-TRF or HFD-TRF+L. **(C)** Daily diet intake, **(D)** daily caloric consumption, **(E)** daily caloric consumption from fat, **(F)** energy efficiency ratio (EER) = body weight gain/caloric consumption during 6 weeks of TRF intervention period x 100. **(G)** Liver mass, **(H)** fasting blood glucose levels, **(I)** fasting insulin, **(J)** insulin resistance index as HOMA-IR after 6 h fasting, **(K)** total fat mass, and **(L)** representative pictures of H&E staining of sections of epididymal adipose tissue are shown. Arrowheads point to crown structures. Scale bar, 200 μm. **(M, N)** Quantitative real-time RT-PCR analysis of selected proinflammatory macrophages (*Adgre1* and *Itgax*) and cytokines (*Tnf*, *Il6*, *Il1b*, *Ifng*) in **(M)** epididymal adipose tissue and **(N)** liver (n=4 per group). **(O)** Leptin levels were assessed by ELISA (n=10 per group). Data are presented as mean ± SEM. Mean values (within the same gene) without a common letter significantly differ at least at *P* < 0.05 by ANOVA with Tukey’s *post hoc* test. n.s., non-significant.

Five-week-old male C57BL/6J mice were purchased from Central Laboratory Animal Inc. (Seoul, Korea) and housed (three to four mice per cage) under controlled temperature (23°C ± 1°C), relative humidity (50% ± 10%) and light/dark cycle (12-h dark/12-h light 7:00 am – 7:00 pm). After 1 week of adaptation, mice were randomly assigned into a low-fat diet (LFD) or high-fat diet (HFD) *ad libitum*. A cohort of mice fed a HFD *ad libitum* for 6 weeks were further divided and maintained another 6 weeks as follows: 1) mice with continued HFD *ad libitum*, mice under time-restricted access to 2) a high-fat diet (HFD-TRF), or 3) a high-fat diet containing 0.005% (w/w) luteolin (HFD-TRF+L) (**Figure 1A**). Diets used in this study are a LFD (D12450B), a 60% HFD (D12492), and a 60% HFD containing 0.005% (w/w) luteolin (D17090604) (all from Research Diet Inc. New Brunswick, NJ, USA). The TRF groups had access to food for 10 h during the dark active phase, from ZT13 to ZT23 where ZT0 denotes light on. Food access was regulated by transferring mice daily between cages with food and water and cages with water only. To control for mouse handling, *ad libitum*-fed mice were also transferred between feeding cages at the same time. Food intake was measured twice a week and body weight was measured once a week. At the end of experiment, blood was collected from tail veins of unanesthetized mice after a 6 h fast (ZT23-ZT5) to measure metabolic phenotype and circulating leukocytes. Immediately after euthanasia, blood was collected by cardiac puncture and organs were harvested at 1 pm (ZT6). All animal experiments were reviewed and approved by the Institutional Animal Care and Use Committee of Chungbuk National University (approval number: CBNUA-1347-20-01 and CBNUA-1602-21-01).

### 2.2 Metabolic parameters

Fasting glucose was obtained from tail veins of mice after 6 h of fasting starting from ZT23 and tested using a Contour glucose meter (Bayer). Fasting insulin concentration was measured from serum, following the manufacturer’s protocol for the Ultra Sensitive Mouse Insulin ELISA kit (Crystal Chem, Downers Grove, IL, USA). Insulin resistance index, HOMA-IR, was calculated as fasting blood glucose (mmol/L) x fasting insulin (mU/L)/22.5 (33). For the leptin ELISA (MOB00, R&D Systems, Minneapolis, USA) measurements, blood serum was diluted 20 to 100 fold. Serum CCL2 level was measured by Mouse CCL2/JE/MCP1 Quantikine ELISA kit (MJE00, R&D systems). Samples were analyzed according to the manufacturers’ instructions.

### 2.3 Histology

Epididymal fat pads were fixed in 4% formaldehyde (Sigma, St. Louis, MO, USA) overnight, embedded in paraffin, sectioned, and stained with hematoxylin and eosin. Digital images were acquired with a Lionheart FX automated microscope (BioTek, Winooski, VT, USA).

### 2.4 Isolation of mouse bone marrow cells

BM from one femur of mice was flushed with PBS (without Mg^2+^ and Ca^2+^) using a syringe, passing through 40-μm cell strainer, and centrifuged at 300 g for 5 min at room temperature. The cell pellets were resuspended in ammonium-chloride-potassium lysing buffer (Invitrogen, Grand Island, NY, USA) on ice for 10 min. Cells were washed and resuspended in 2% FBS/PBS for flow cytometry analysis or washed with PBS, and then pellets were collected for RNA isolation and real-time PCR. BM hematopoietic stem and progenitor cells (HSPC) were enriched using an EasySep™ Mouse Hematopoietic Progenitor Cell Isolation kit (STEMCell Technologies, Vancouver, Canada), following the manufacturer’s procedures. Isolated HSPC were used for RNA isolation and real-time PCR.

### 2.5 Flow cytometry

#### 2.5.1 Hematopoietic stem cells and myeloid progenitors in BM

BM cells prepared above were stained with lineage markers on FITC (CD4, CD5, CD8, CD11b, B220, Gr1, Ter119, and CD41, eBioscience, San Diego, CA, USA), CD117-APC-H7 (BD Bioscience, San Jose, CA, USA), Sca1-PE (eBioscience), CD16/32-PerCP5.5 (BD Bioscience), CD150-PE-Cy7 (BioLegend, San Diego, CA, USA), and CD105-BV421 (BD Bioscience) for 30 min on ice and gating as described by Pronk et al (34). and Singer et al. (12). Dead cells were excluded using 7-Amino-Actinomycin D (7-AAD; eBioscience) and AccuCheck counting beads (PCB100, Invitrogen) were used for quantification of absolute cell numbers. Multiparameter analysis was performed on a FACSSymphony A3 (BD Bioscience) using the FACSDiva software and data were analyzed using the FlowJo software (version 10, Tree Star Inc., Ashland, OR, USA).

#### 2.5.2 Mature immune cells in BM

BM cells were incubated with Fc Block (BD Bioscience) for 15 min, and then stained with the following conjugated antibodies (30 min at 4°C in the dark): CD45-APC-Cy7, NK1.1-APC, CD3-APC, CD19-APC, CD11b-PerCP-Cy5.5, Ly6C-FITC (all from BD Bioscience), TER119-APC, Ly6G-eFluor450 (eBioscience) for neutrophil and monocyte populations, CD45-APC-Cy7, CD19-PE, CD4-V500, CD8-PerCP-Cy5.5, Gr1-APC (BD Bioscience), and CD3-eFlour450, NK1.1-PE-Cy7, Ter119-APC (eBioscience) for lymphocyte population. After antibody staining, the cells were incubated with 7-AAD for 5 min at room temperature as a viability dye for dead cell exclusion and analyzed in the presence of AccuCheck counting beads by FACSSymphony A3. The data were further analyzed by using the FlowJo software.

#### 2.5.3 Blood leukocytes

Blood was collected from tail veins in the presence of 5 mM EDTA, incubated with BD Fc Block for 10 min, stained with mAbs specific to CD45 (clone 30-F11, BD Bioscience), CD11b (M1/70, BD Bioscience), Ly6C (AL-21, BD Bioscience), Ly6G (1A8, eBioscience), NK1.1 (PK136, eBioscience), CD3 (17A2, eBioscience), CD19 (1D3, BD Bioscience), TER119 (TER119, eBioscience), CD4 (RM4-5, BD Bioscience), CD8 (53-6.7, BD Bioscience), Gr1 (RB6-8C5, BD Bioscience) for 20 min at room temperature. The blood was then fixed and lysed using BD FACS Lysing solution (BD Bioscience). After fixation, cells were washed and resuspended in 2% FBS/PBS and then immediately analyzed by FACSSymphonyA3 and FlowJo software. AccuCheck counting beads were used for quantification of absolute cell numbers. Gating strategy for counting beads is shown in **Supplementary Figure S1**.

### 2.6 *In vivo* proliferation assay

Mice were injected intraperitoneally with 40 mg/kg 5-ethynyl-2’-deoxyurine (EdU, Sigma) dissolved in PBS (35, 36). BM was collected 18 h later, and EdU incorporation was determined by Click-iT EdU Flow Cytometry Assay kits (Invitrogen) according to the manufacturer’s protocol. Briefly, BM cells were stained with FITC-conjugated antibodies for lineages (CD4, CD5, CD8, CD11b, B220, Gr1, Ter119, and CD41) as well as CD117-APC-H7 in 1% BSA/PBS for 30 min on ice. Labeled cells were fixed, permeabilized, and stained with Alexa Flour 647-conjugated azide using Click-iT system. They were then washed and stained with Sca1-PE. Proliferation was quantified and expressed as percentage of EdU^+^ cells.

### 2.7 Real-time PCR

Total RNA from adipose tissue or liver was extracted using the RNeasy Lipid Mini Kit (Qiagen, Palo Alto, CA, USA) and from BM cells or BM HSPC using the NucleoSpin RNA XS kit (Takara Bio, Shiga, Japan), according to the manufacturers’ protocols. First-strand cDNA was synthesized using the Advantage RT for PCR kit (Clontech, Palo Alto, CA, USA). TaqMan gene expression assays were used to quantify target genes, using TaqMan Universal PCR Master Mix (Applied Biosystems, Foster City, CA) and primers for *Adgre1* (Mm00802529_m1), *Itgax* (Mm00498698_m1), *Tnf* (Mm00443258_m1), *Il6* (Mm00446190_m1), *Il1b* (Mm00434228_m1), *Ifng* (Mm01168134_m1), *Ccl2* (Mm00441242_m1), *Ccl8* (Mm01297183_m1), *Spi1* (PU.1, Mm00488140_m1), *Pax5* (Mm00435501_m1), *Gfi1* (Mm00515853_m1), *Cebpa* (Mm00514283_s1), *Lepr* (Mm00440181_m1) and *Tbp* (Mm00446973_m1)(all FAM probes, Applied Biosystems). Samples were run on a QuantStudio 5 Real-Time PCR system (Applied Biosystems) and the fold changes were calculated as 2^-ΔΔCt^ compared with the endogenous control gene, TATA box binding protein (TBP) using LFD as the reference group.

### 2.8 Statistics

Data are presented as mean±SEM. Significant differences among groups were determined by ANOVA followed by Tukey’s HSD *post hoc* procedure. All statistical analysis were performed using SPSS software (version 25.0, SPSS, Chicago, IL, USA), with P values smaller than 0.05 considered significant.

## 3 Results

### 3.1 Effects of time-restricted feeding on obesity and associated parameters

First, we determined the therapeutic effects of 10 h-TRF, alone or in combination with luteolin supplementation, on body weight gain, adiposity, and other metabolic parameters. To induce overweight and obesity, mice were placed on an HFD *ad libitum* for 6 weeks and reached body weight over 40 g on average. After mice on TRF groups were switched to a HFD-TRF or HFD-TRF+L regimens, they weighed significantly less than *ad libitum* group (HFD-AL) throughout the study (**Figure 1B**). At the end of experiment, the body weights of the HFD-TRF and HFD-TRF+L were 17.1% and 18.5% lower than the HFD-AL group. The average intake in diet, calorie, and fat calorie in the HFD-AL group were significantly higher than that in the HFD-TRF and HFD-TRF+L groups (**Figures 1C–E**). Additionally, we calculated the energy efficiency ratio (EER) to assess the efficiency of animals in converting energy consumption into increased body weight. We found that EER was significantly higher in the HFD-AL group than others, while mice in the HFD-TRF or HFD-TRF+L group exhibited lower EER values than those in the LFD *ad libitum* (LFD-AL) group (**Figure 1F**), even with similar caloric intake and higher fat consumption. However, there was no significant difference between HFD-TRF and HFD-TRF+L groups. These results indicate that TRF intervention may be helpful for weight management beyond controlling dietary intake.

Similarly, TRF intervention significantly reduced liver mass, fasting blood glucose, insulin, and insulin resistance index HOMA-IR (**Figures 1G–J**). Despite similar total fat mass among HFD-fed groups (**Figure 1K**), H&E stained sections of epididymal adipose tissue showed characteristic crown-like structures in HFD-AL mice that were absent in both HFD-TRF and HFD-TRF+L mice (**Figure 1L**). In parallel, we found that HFD *ad libitum* resulted in a significant elevation of macrophage-associated genes *Adgre1* (F4/80) and *Itgax* (CD11c) and proinflammatory cytokines *Tnf and Il6*, while TRF dramatically decreased them in adipose tissue (**Figure 1M**) as well as liver (**Figure 1N**). In addition, mice under HFD-TRF intervention exhibited less leptin in circulation when compared with those under HFD-AL (**Figure 1O**). To summarize, TRF exerts therapeutic effects in overweight and obese mice by lowering body weight gain and liver mass. It protected against adipose tissue inflammation as well as insulin resistance associated with obesogenic diets. However, luteolin supplementation showed no additive effect compared with TRF alone on these metabolic parameters (**Figures 1B–O**), except *Adgre1* (F4/80) mRNA expression in adipose tissue (**Figure 1M**).

### 3.2 Effects of time-restricted feeding on obesity-induced switch from quiescent to differentiating LSK cells

**Figure 2:**
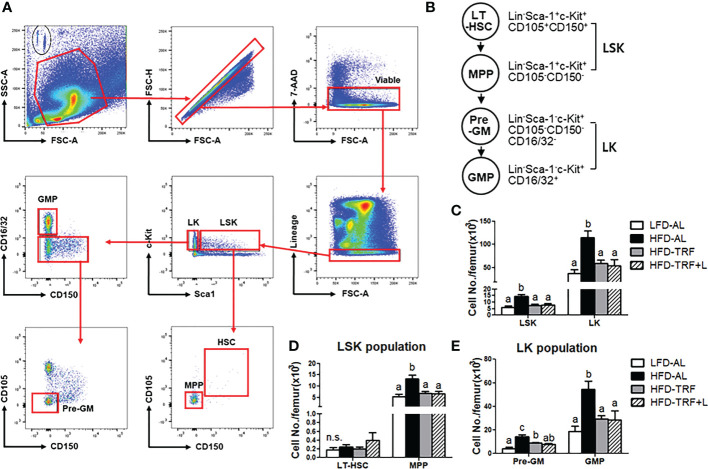
Time-restricted feeding (TRF) normalizes obesity-induced myeloid progenitors. BM were obtained in mice (n=7 per group) after 12 weeks of LFD *ad libitum* (LFD-AL), HFD *ad libitum* (HFD-AL), HFD *ad libitum* followed by HFD but TRF intervention (HFD-TRF), or HFD *ad libitum* followed by HFD supplemented with luteolin but TRF intervention (HFD-TRF+L). **(A)** Gating strategy for hematopoietic stem cells using singlets, viable, lineage negative cells and then LSK and LK populations for sub-gating of LT-HSC, MPP, Pre-GM, and GMP cells. **(B)** Schematic overview of HSPC maturation in the BM. **(C)** Flow cytometry quantification of bone marrow LSK and LK cells, **(D)** LT-HSC and MPP, and **(E)** pre-GM and GMP. Data are presented as mean ± SEM. Means without a common letter significantly differ at least at *P*<0.05 by ANOVA with Tukey’s *post hoc* test. HSPC, hematopoietic stem and progenitor cells; LT-HSC, long term hematopoietic stem cells; MPP, multipotent progenitors; Pre-GM, pre-granulocyte macrophage progenitor; GMP, granulocyte-macrophage progenitor. n.s., non-significant.

HSPC are defined as Lin^-^Sca-1^+^c-Kit^+^ (LSK) cells (12, 34, 37), and within the LSK population, there are different subsets, including long-term hematopoietic stem cells (LT-HSC; Lin^-^Sca-1^+^c-Kit^+^CD150^+^CD105^+^) and multipotent progenitors (MPP; Lin^-^Sca-1^+^c-Kit^+^CD150^-^CD105^-^) (**Figures 2A, B**). LT-HSC are capable of self-renewal, but this characteristic is lost when they mature and differentiate to MPP. Our results showed that 12 weeks of HFD *ad libitum* increased the number of LSK cells in the BM, compared to those under LFD (**Figure 2C**). Interestingly, within the LSK population, HFD *ad libitum* increased MPP population whereas the LT-HSC was not affected (**Figure 2D**). Furthermore, mice under same obesogenic diet but with TRF intervention, exhibited smaller numbers of LSK and MPP cells, similar to a level seen in those with LFD. Together, our data indicate that HFD *ad libitum* shifts the characteristics of HSPC from a quiescent population capable of self-renewal toward a more mature HSPC population with higher differentiation potential; these alterations caused by an obesogenic diet could be protected by TRF intervention.

Differentiating LSK cells loose Sca-1 and/or c-Kit, becoming lineage-committed progenitors [myeloid progenitor Lin^-^c-Kit^+^ cells (LK), lymphoid progenitor Lin^-^Sca-1^+^ cells (LS)] (12, 34, 37). LK cells start to express markers for granulocyte macrophage progenitors (GMP; Lin^-^Sca-1^-^c-Kit^+^CD16/32^+^). Along with a shift within the LSK population toward a more mature HSPC population with higher differentiation potential, there was a significant increase in LK population by HFD, which were normalized by TRF intervention (**Figure 2C**). Within the LK population, HFD *ad libitum* increased both pre-GM and GMP numbers and TRF intervention decreased it by ~50% (**Figure 2E**). These data suggest that HFD *ad libitum* could induce differentiation of BM cells into mature myeloid cells, which could be restored by TRF intervention. There was no additive effect of luteolin when administered along with TRF intervention.

### 3.3 Effects of time-restricted feeding on obesity-induced immune cell profile in the peripheral blood

**Figure 3:**
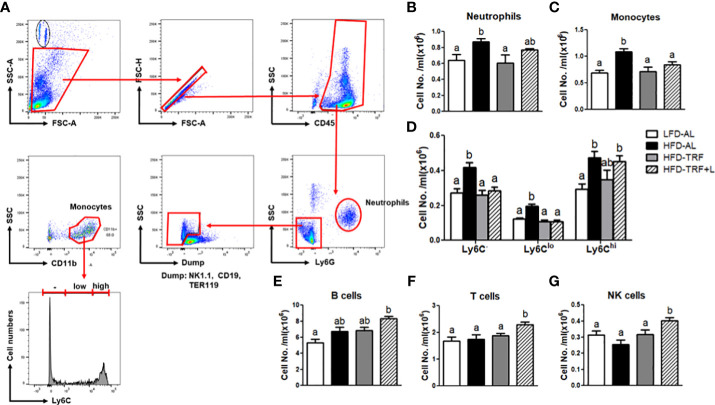
Obesity increases the numbers of circulating neutrophils and monocytes, and time-restricted feeding (TRF) reverses these increases. **(A)** Gating strategy for flow cytometric analysis of circulating neutrophils and monocytes. Absolute numbers of **(B)** neutrophils (CD45^+^Ly6G^+^), **(C)** monocytes (CD45^+^NK1.1^-^CD19^-^TER119^-^CD11b^+^), **(D)** monocyte subpopulation based on Ly6C expression levels, **(E)** B cells (CD45^+^Gr1^-^TER119^-^CD19^+^CD3^-^), **(F)** T cells (CD45^+^Gr1^-^TER119^-^CD19^-^CD3^+^), and **(G)** NK cells (CD45^+^Gr1^-^TER119^-^CD19^-^CD3^-^NK1.1^+^) were measured in the blood of mice (n=7 per group) after 12 weeks of LFD *ad libitum* (LFD-AL), HFD *ad libitum* (HFD-AL), HFD *ad libitum* followed by HFD but TRF intervention (HFD-TRF), or HFD *ad libitum* followed by HFD supplemented with luteolin but TRF intervention (HFD-TRF+L). Data are presented as mean ± SEM. Means without a common letter significantly differ at least at *P*<0.05 by ANOVA with Tukey’s *post hoc* test.

GMP can generate mainly neutrophils, monocytes, and macrophages and a minor population of eosinophils, basophils, and mast cells (38). Thus, we investigated whether *ad libitum* or TRF of a HFD could alter immune cell profile, including neutrophils and monocytes in circulation by flow cytometric analysis (**Figure 3A**). We found that HFD *ad libitum* increased the number of Ly6G^+^ neutrophils and CD11b^+^ monocytes (**Figures 3B, C**) and TRF intervention of a HFD normalized these numbers to the numbers seen in the LFD *ad libitum* group. We further examined the effect of HFD *ad libitum* or TRF on the changes in monocyte subpopulation. HFD *ad libitum* led to a significant induction of Ly6C^-^ and Ly6C^lo^ patrolling monocytes as well as pro-inflammatory Ly6C^hi^ monocytes (**Figure 3D**), whereas TRF intervention significantly decreased Ly6C^-^ and Ly6C^lo^ subpopulation and tended to lower circulating Ly6C^hi^ monocytes (p=0.111). We extended our search to other immune cells; neither obesity nor TRF affect B cells, T cells, and natural killer (NK) cells in circulation (**Figures 3E-G**). Unexpectedly, TRF in combination with luteolin exhibited a slight different phenomenon. TRF with luteolin decreased the numbers of the Ly6C^-^ and Ly6C^lo^, resulting in a decrease in monocyte numbers similar to TRF alone (**Figures 3C, D**), but it increased the numbers of B cells, T cells, and NK cells compared to the LFD-AL group (**Figures 3E-G**), which remains to be further investigated. These data suggest that HFD *ad libitum* increased the numbers of circulating neutrophils and monocytes, and that TRF intervention by itself was sufficient to normalize these systemic changes.

### 3.4 Effects of time-restricted feeding on mature neutrophils and monocytes in BM

**Figure 4:**
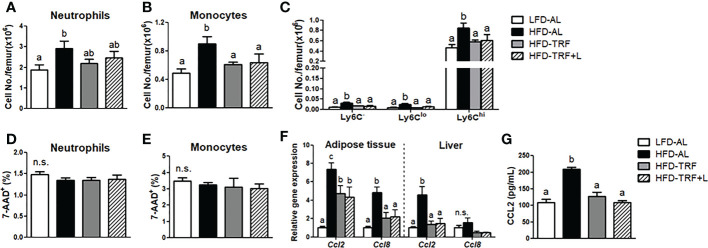
Effects of Time-restricted feeding (TRF) on mature neutrophils and monocytes in BM. Absolute numbers of **(A)** neutrophils (CD45^+^Ly6G^+^), **(B)** monocytes (CD45^+^NK1.1^-^CD19^-^TER119^-^CD11b^+^) and **(C)** monocyte subpopulation based on Ly6C expression levels as well as frequency of 7-AAD^+^ cells among **(D)** neutrophils and **(E)** monocytes were measured in the BM of mice (n=7 per group) after 12 weeks of LFD *ad libitum* (LFD-AL), HFD *ad libitum* (HFD-AL), HFD *ad libitum* followed by HFD but TRF intervention (HFD-TRF), or HFD *ad libitum* followed by HFD supplemented with luteolin but TRF intervention (HFD-TRF+L). **(F)** Quantitative real-time RT-PCR analysis of selected chemokine (*Ccl2* and *Ccl8*) in epididymal adipose tissue (n=8 per group) and liver (n=4 per group). **(G)** Serum CCL2 levels were assessed by ELISA (n=10 per group). Data are presented as mean ± SEM. Mean values (within the same gene and tissue) without a common letter significantly differ at least at *P*<0.05 by ANOVA with Tukey’s *post hoc* test. n.s., non-significant.

Decreased numbers of circulating monocytes and neutrophils could be due to not only reduced BM myelopoiesis but also increased myeloid cell death in the BM. First, we analyzed the mature neutrophils and monocytes in the BM. Ly6C^hi^ monocytes represented roughly 95% of BM monocytes. We detected an increased number of mature neutrophils and monocytes by HFD *ad libitum* (**Figures 4A-C**). We also detected decreased numbers of BM monocytes, including Ly6C^hi^ and a tendency towards decreased BM neutrophils by TRF intervention (**Figures 4A-C**). Furthermore, the frequency of dead (7-AAD^+^) neutrophils or monocytes was similar in the BM, regardless of diet or TRF intervention (**Figures 4D, E**), suggesting that TRF intervention did not particularly promote myeloid cell death in the BM. In mice under TRF, adipose tissue and liver produce less CCL2, a critical chemokine for monocyte mobilization from the BM (39)(**Figure 4F**), leading to decreased levels of chemokine in blood (**Figure 4G**). Thus, less supply of monocytes and CCL2 may cause less monocytes in circulation.

### 3.5 Time-restricted feeding decreases gene expression of transcription factors required for myeloid differentiation in BM without affecting BM progenitor cell proliferation

**Figure 5:**
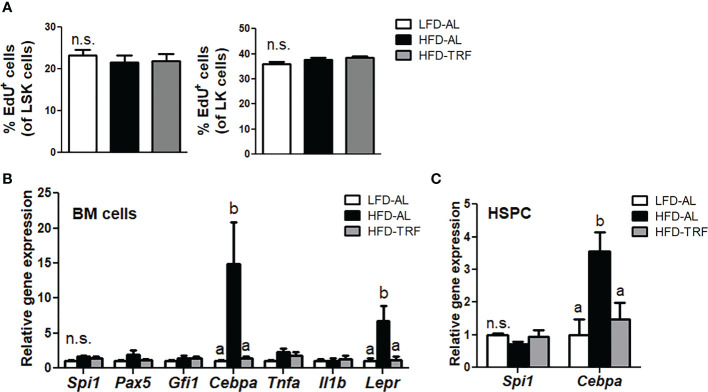
Time-restricted feeding (TRF) decreases gene expression of transcription factor required for myeloid differentiation without affecting BM progenitor cell proliferation. C57BL/6 mice were fed an LFD *ad libitum* (LFD-AL), HFD *ad libitum* (HFD-AL), HFD *ad libitum* followed by HFD but TRF intervention (HFD-TRF) for 12 weeks. **(A)** EdU incorporation in LSK cells (Left) or LK cells (Right), as measured 18 h after intraperitoneal injection (n=8 per group). **(B)** Quantitative real-time RT-PCR analysis of selected hematopoietic transcription factors (*Spi1, Pax5, Gfi1, Cebpa*), cytokines (*Tnfa* and *Il1b*), and leptin receptor (*Lepr*) in BM cells (n=6 per group). **(C)** Quantitative real-time RT-PCR analysis of *Spi1* and *Cebpa* in BM HSPC (n=5 per group). Data are presented as mean ± SEM. Mean values (within the same gene) without a common letter significantly differ at least at *P*<0.05 by ANOVA with Tukey’s *post hoc* test. n.s., non-significant.

Since we observed changes in myeloid progenitors and mature monocytes in the BM by TRF, we performed *in vivo* EdU labeling in mice to determine the proliferative capacity of LSK cells in the BM. Interestingly, LSK as well as LK cells exhibited similar proliferation rate in HFD-AL group compared with LFD-AL group (**Figure 5A**). In addition, TRF intervention did not cause a significant difference in EdU incorporation in both LSK and LK cells, suggesting myeloid differentiation, not proliferation of progenitors, may contribute to changes in myeloid cell population.

Myeloid differentiation fate is determined by lineage-specific transcription factors (40). Among them, the transcription factor C/EBPα functions in self-renewal of HSC and drives myeloid differentiation, indispensable for GMP generation (40). We found that HFD *ad libitum* resulted in a significant elevation of *Cebpa* mRNA levels in BM cells, where TRF intervention led to the level similar to that seen in the LFD-AL group (**Figure 5B**). However, expression of other transcription factor genes, including *Pax5* required for lymphocyte differentiation (41), was unaltered. In addition, expression of *Tnf* and *Il-1b*, cytokines that have been shown to activate HSC (42), did not differ among LFD-AL, HFD-AL, and HFD-TRF groups. Interestingly, HFD *ad libitum* increased *Lepr* expression, known to be an important factor for maintaining BM homeostasis (43), in BM cells by 6.8-fold, where TRF decreased by ~80%. Furthermore, changes in *Cebpa* mRNA levels by HFD and TRF were also observed in BM HSPC (**Figure 5C**). These data suggest that TRF may influence myeloid development through balancing transcription factors required for differentiation at the undifferentiated progenitor cell stage.

## 4 Discussion

**Figure 6:**
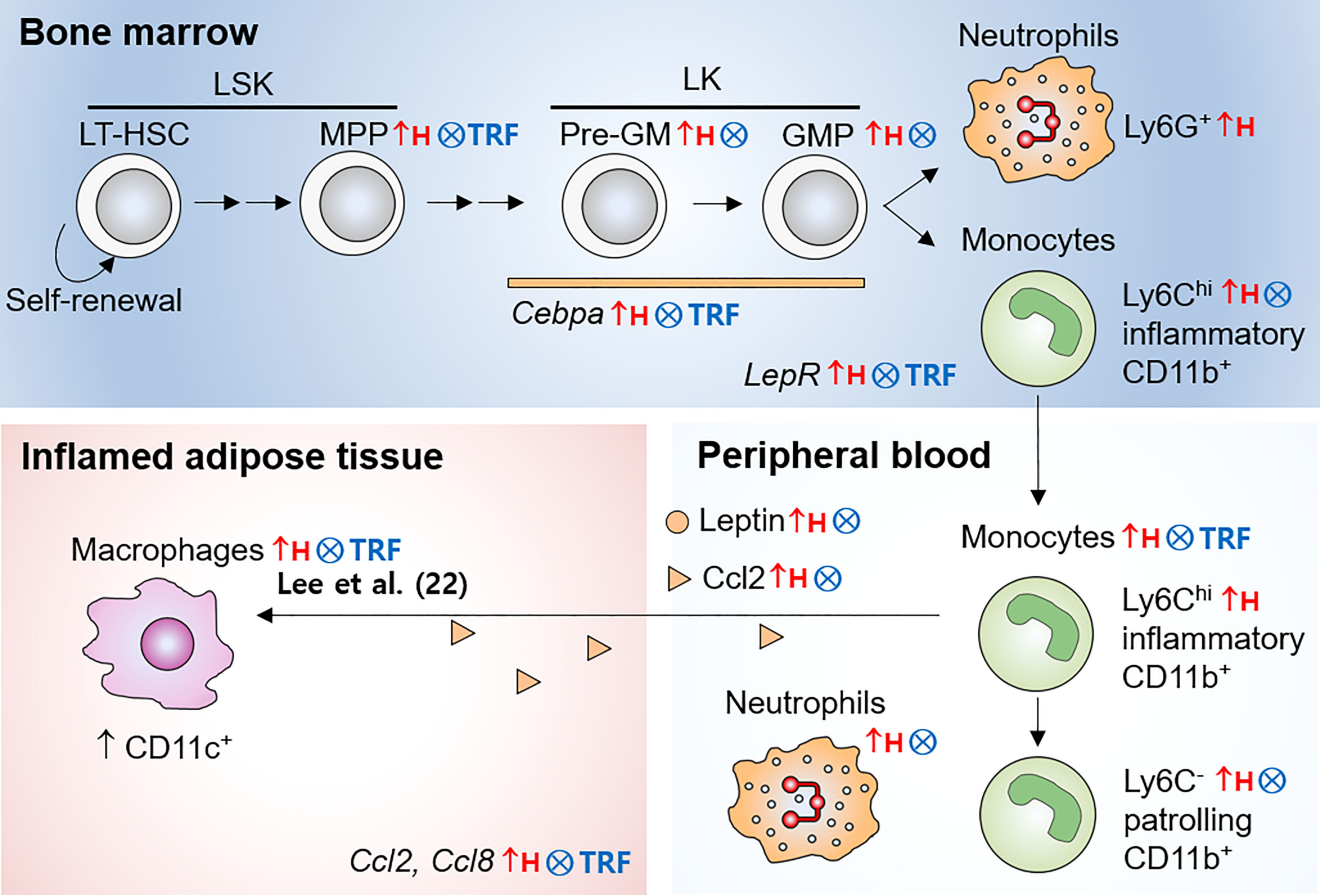
Illustration of monocyte pools influenced by obesity and TRF intervention. In the bone marrow, LT-HSC differentiate into short-term HSC, and subsequently to MPP with reduced self-renewal ability. MPP differentiate into Pre-GM and others. Pre-GM give rise to GMP, and subsequently generates monocytes and neutrophils. While HFD *ad libitum* led to expansion of multiple early myeloid precursor cells, such as MPP, Pre-GM, and GMP, TRF inhibited these increases. In parallel, TRF reduced the numbers of mature monocyte in the bone marrow and peripheral blood. TRF was effective to reduce leptin in circulation and *LepR* mRNA expression in BM cells. In addition, TRF reduced mRNA expression of the transcription factor *Cepba* in BM cells. In summary, TRF is an effective dietary strategy to reduce overproduction of monocytes during obesity, which may be associated with the reduced CD11c^+^ adipose tissue macrophages and insulin resistance seen in our previous study (22). LT-HSC, long-term hematopoietic stem cells; MPP, multipotent progenitors; Pre-GM, pre-granulocyte macrophage progenitors; GMP, granulocyte macrophage progenitors; LSK, Lin^-^Sca-1^+^c-Kit^+^; LK, Lin^-^c-Kit^+^ cells; Lepr, leptin receptor; ↑H, increased by HFD *ad libitum*; ⨂, inhibited by TRF intervention.

Obesity is associated with infiltration of immune cells, notably macrophages, into adipose tissue, which may contribute to systemic insulin resistance (4, 5). Since the production of these cells can influence systemic cell abundance (44) and recruitment to the adipose tissue (12), using dietary intervention that reduces overproduction of monocytes may provide the strategies to reduce insulin resistance associated with obesity. As summarized in **Figure 6**, TRF normalized obesity-induced expansion of multiple early myeloid progenitor populations in the BM. In parallel, TRF reduced the monocyte pool in the BM and in circulation to a level similar to that seen in mice fed LFD *ad libitum*. Along with these changes, mice on TRF were also protected against insulin resistance. In sum, effect of TRF on HSPC phenotype that is mirrored at neutrophils and monocytes in circulation could in part explain the systemic health improvements seen under TRF.

How does TRF alter monocyte supply? First, we demonstrated decreased mRNA expression of transcription factor, important for myeloid differentiation, such as *Cebpa* in the BM HSPC. The establishment of hematopoietic lineages is orchestrated by a hierarchical network of transcription factors, each of which are essential for specific steps (40). C/EBPα functions in self-renewal of existing hematopoietic stem cells and has indispensable role in GMP generation (40). Growth-factor independent 1 (GFI1) is required for HSC self-renewal and late-stage neutrophil production (40). PU.1 expression seems to be essential in both myeloid and lymphoid progenitors (40). Among them, only *Cebpa* mRNA expression was upregulated by HFD *ad libitum* and normalized by TRF. While HFD *ad libitum* induced the quantitative changes in multiple early myeloid progenitor populations, including MPP in LSK population, Pre-GM and GMP in LK population, TRF intervention normalized them. However, LSK and LK cells exhibited similar proliferative capacity, regardless of diet or TRF intervention. In a study, using a cell-tracing (*in vivo*) approach, the authors found that hematopoietic stem cells were able to differentiate into MPP and even restricted myeloid progenitors, without undergoing cell division (45). Therefore, TRF appears to reduce differentiation of HSPC in favor of myelopoiesis during obesity rather than influencing proliferation rate; *Cepba* expression level may play a crucial role in this process.

Further analysis in immune cell profile and 7-AAD^+^ frequency in BM indicates that TRF-induced reduction in the numbers of BM monocytes was not due to increased cell death. We found that TRF mice had lower levels of circulating CCL2, one of chemokines that is primarily responsible for CCR2 activation and monocytes mobilization (39). While CCL2/CCR2 axis on myeloid cells remains to be investigated further, these data suggest that reduced supply of monocytes and CCL2 by TRF intervention may contribute to reduced number of monocytes in circulation.

There are several lines of evidence suggesting that metabolic and immunologic effect of TRF may be interrelated. First, TRF effectively reduced glucose levels in overweight and obese mice (**Figure 1H**). Similar result was found in meta-analysis with 238 participants, particularly who had metabolic abnormality (46). Interestingly, streptozotocin or Akita diabetic mice were shown to have increased numbers of circulating monocytes and neutrophils but not lymphocytes, along with enhanced myelopoiesis in the BM, and these changes were normalized by lowering glucose levels (47). Similarly, *Apoe* ^-/-^ mice exposed to hyperglycemia (4 injection of 2 g/kg glucose, 2 h apart) once a week for 10 week showed similar myelopoiesis likely contributing to monocytosis and accelerated atherosclerosis (48). In the current study, we observed that 6 weeks of daily 10 h-TRF intervention led to normoglycemia similar to the LFD group despite of HFD consumption. Thus, the protective role of TRF may be in part mediated through preventing or lowering HFD-induced hyperglycemia, which has been linked to hematopoietic disruption and particularly to myeloid skewing.

Second, TRF effectively modulated leptin levels in circulation (**Figure 1O**). It is well known that plasma leptin levels decrease during fasting or energy restriction and increase when energy is abundant (49). Chaix and others reported that serum leptin levels were lower in mice on HFD with TRF intervention, compared to HFD *ad libitum* (17, 50). We also confirmed that HFD *ad libitum* resulted in a significant elevation of leptin levels in circulation (3.5-fold) compared to LFD group, where TRF decreased them by ~30%. Interestingly, we found that *Lepr* expression in BM cells was high in mice under HFD *ad libitum* and reduced by TRF intervention (**Figure 5B**). Although only a minor subset of long-term HSC express *Lepr* under steady state, Lepr^+^ stromal cells are known to play critical roles in maintaining BM homeostasis (43). A recent study reported involvement of leptin signaling in hematopoietic output by modulating BM stromal cells in the context of exercise (51). While voluntary exercise reduced serum leptin levels and LSK cell proliferation, leptin supplementation during exercise increased numbers of leukocytes (51). Hence, it is plausible that TRF exerts protection *via* lowering and maintaining leptin levels due to prolonged fast, which may link to the reduced hematopoietic output of monocytes and neutrophils.

Most importantly, TRF was able to reduce peripheral monocytes in overweight and obese mice (**Figure 3C**). Obesity-induced increase in circulating monocytes has been shown in both mice (52) and humans (6). Monocytes rapidly migrate during obesity and they to a greater extent infiltrate into inflamed adipose tissue to become ATM (53). Since ATM are associated with obesity-associated insulin resistance (11, 54–56), reduced numbers of monocytes by TRF may result in fewer ATM and perhaps also with less proinflammatory profile for these ATM. Indeed, we previously reported that TRF intervention reduced numbers of total and proinflammatory ATM, along with the improvement of insulin resistance in overweight and obese mice (22). Real-time PCR of the total adipose tissue for the macrophage-associated genes *Adgre1* (F4/80) and *Itgax* (CD11c) also indicated that TRF decreased proinflammatory ATM infiltration (**Figure 1M**), consistent with our previous findings (22) (**Figure 6**). Thus, the regulation of monocyte pools by TRF may be a contributing factor to alleviated insulin resistance associated with obesity.

In our experiment, we applied TRF intervention to mice during the active (dark) phase. However, not all TRF regimens are beneficial. In fact, TRF out of sync with the circadian timing was shown to worsen glucose intolerance (21) and increase diet-induced obesity (57). It is noteworthy that mice fed only in the active (dark) period had increased energy expenditure, compared with those fed only in the inactive (light) period (58). This may in part explain the lower efficiency of TRF mice in converting energy consumption into body weight, even with LFD-AL despite similar caloric intake. These results suggest that TRF may be effective for having metabolic benefits when administered in line with the circadian timing.

The current study also explored the effects of TRF and concurrent luteolin supplement on metabolic parameters and hematopoiesis. Since luteolin has been shown to be effective in reducing obesity and obesity-associated inflammation in previous studies (29–32), we expected that TRF in combination with luteolin supplementation would be more effective than TRF alone. However, we found no significant difference between the two treatments in terms of body weight, adiposity, liver mass, insulin resistance, and leptin level. In addition, there was no additive effect of luteolin on the improvement against myelopoiesis, neutrophilia, and monocytosis, when compared with TRF alone. Unexpectedly, mice under HFD-TRF+L had more Ly6C^hi^, B cells, T cells, and NK cells than those under LFD-AL. These results are difficult to interpret. We could only speculate that increased lifespan of these cells or increased inflammation might take place when luteolin is combined with TRF. However, we did not see adipose tissue inflammation induced by TRF in combination with luteolin, compared with TRF alone. Thus, until functional comparisons of these peripheral immune cells are confirmed, the interpretation and implication of these results need to be addressed carefully. Furthermore, we focused on the changes of certain metabolic and immunologic aspects and thus, there are possibility that luteolin may deliver other benefits in combination with TRF, which needs to be determined in the studies with varying dose and duration.

A potential limitation of this study is that small amount of RNA in HSPC is a severe limiting factor which deprived us of the ability to compare among a wide range of transcriptional genes and cytokines needed for hematopoiesis. It would be advisable to perform single cell RNA-sequence to elaborate the effect of TRF in lineage specification and gene regulatory networks of distinct HSPC clusters.

In summary, we in this study demonstrated that TRF intervention effectively reduced BM myeloid progenitors, including MPP, Pre-GM, and GMP leading to normalization of monocyte numbers in both BM and blood, while without affecting circulating B, T, and NK cells. These results suggest a great potential for using TRF regime to counteract obesity-induced disturbance in BM hematopoiesis, and this effect may in part contribute to health benefits of practicing TRF, in particular increasing insulin sensitivity.

## Data availability statement

The original contributions presented in the study are included in the article/**Supplementary Material**. Further inquiries can be directed to the corresponding author.

## Ethics statement

The animal study was reviewed and approved by the Institutional Animal Care and Use Committee of Chungbuk National University.

## Author contributions

MP conceived and designed the study. YK, YL, MNL, JN, NY and MP performed the experiments. YK and MP analyzed and interpreted the data. YK, MNL, DW, and MP wrote, edited, and reviewed the manuscript. MP had full access to all data in the study and took responsibility for the integrity of the data, and also for the manuscript. All authors listed have made a substantial, direct, and intellectual contribution to the work and approved it for publication.
