# Supplementary figures and images for: Time-restricted feeding reduces monocyte production by controlling hematopoietic stem and progenitor cells in the bone marrow during obesity
Source: Front Immunol. 2022 Dec 8;13:1054875. doi: 10.3389/fimmu.2022.1054875 (PMC9771705; doi:10.3389/fimmu.2022.1054875)

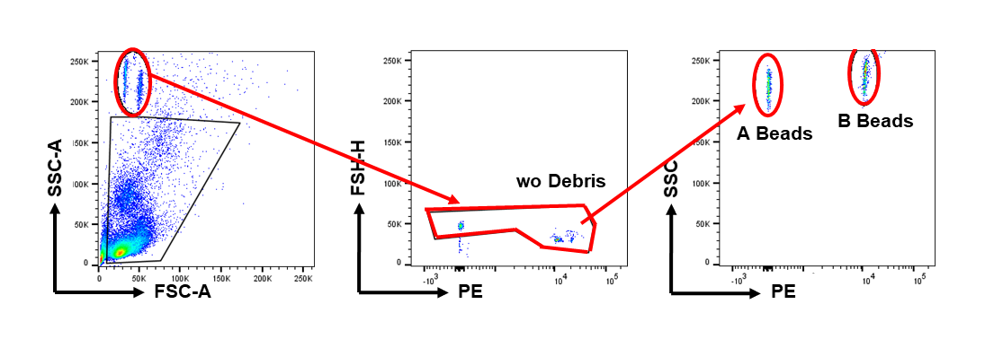


**Supplementary Figure S1.** Gating strategy for counting beads

Supplement: Supplementary file 1 [file DataSheet_1.docx]
